# Aneurism of the Aorta

**Published:** 1827-08

**Authors:** 


THE LONDON
### Medical and Physical Journal.

N°. 342, VOL. LVIII.] AUGUST, 1827. [N° 14, *New Series.*

For many fortunate discoveries in medicine, and for the detection of numerous errors, the world is indebted to the rapid circulation of Monthly Journals; and there never existed any work, to which the Faculty, in Europe and America, were under deeper obligations, than to the Medical and Physical Journal of London, now forming a long, but an invaluable, series.—RUSH.

### ORIGINAL PAPERS,

AND

CASES OBTAINED FROM PUBLIC INSTITUTIONS AND OTHER AUTHENTIC SOURCES.

### ANEURISM OF THE AORTA.

In our last Number we gave some cases of rupture of the aorta without aneurism: we now subjoin some cases illustrating the phenomena presented by aneurism of that vessel terminating in rupture. Perhaps, the circumstance most worthy of remark is the absence of any considerable inconvenience till within a very short period of death. In the first case, the patient (who was of the highest rank in the Spanish army, and made a very distinguished figure during the early part of the Peninsular campaign,) led a life of constant bodily and mental exertion.

The subject of the second case followed a laborious profession to the last, and, up to the hour of his death, took exercise of a kind requiring great exertion, which he accomplished without any apparent difficulty. It is, perhaps, worthy of remark, as connected with his case, that he was in the habit of doing this very frequently during the last two years of his life.

The third case is remarkable from having assumed the appearance of stricture of the œsophagus, and from the rupture taking place into that tube, so as to fill the stomach with blood.

In our Collectanea will be found a fourth case, from an American Journal, in which aneurism of the abdominal aorta burst into the intestinal canal.

### *Case of Aneurism of the Aorta which burst into the Pericardium.*

By T. Rose, Esq.

On the 17th January, 1811, a Spanish officer of distinction, whilst walking through a room in his quarters at Cartaxo, after breakfast, was suddenly attacked with most excruciating pains darting through his chest, attended with a sense of suffocation and great difficulty of breathing. I was sent for, and saw him in half an hour after he was taken ill. I found him in bed, restless and alarmed, breathing short, with the greatest anxiety, and unable to distend the lungs, or even to attempt doing so. His pulse was about 110. The pains shooting through his chest had somewhat abated before I got to him, but were still very severe. By quieting his mind, the oppressive feelings and difficulty of breathing began to wear off. Towards the afternoon, and during the whole of the night, he perspired freely, and all the symptoms gradually left him.

He had never had any similar attack, and his health previous to the present one had been excellent. He was constantly taking violent exercise, without pain or inconvenience; had never been affected by complaints in his chest, by palpitations about his heart, nor by any thing which could at all assist me in ascertaining the cause of the severe attack he had just experienced.

As his bowels were very costive, and had been so for several days, I ordered him some opening medicines, and watched carefully for any further symptoms which might occur.

He had slight exacerbations of fever towards evening, for two or three days after the 17th, attended with a good deal of pain in his stomach and bowels, which were moved with great difficulty. On free evacuations being procured, all these pains left him, and he was gradually gaining strength.

On the evening of the 20th, I observed, for the first and only time, an irregularity in his pulse: it intermitted every second beat. He had slight headache that evening; and he informed me next day that he had felt, during the early part of the night, a good deal of what he called rheumatic pain, shooting from his chest towards the insertions of the pectoral muscles. In the latter part of the night he slept well, and was better on the 21st than on any preceding day. A physician, who had resided in his family for some time, assured me that no intermission in his pulse had ever before been observed. I was the more particular in my inquiries into this, as I could only account for the symptoms in his late attack by supposing it to have arisen from some obstruction to the circulation, in or about the heart.

He sat up for a short time on the 21st and 22d; and on the 23d he was so far recovered that he proposed walking in the course of the day to head-quarters, which were at the extremity of the village. I sat with him for nearly an hour that morning. He was in excellent spirits; said his appetite was good, and that he felt himself considerably stronger than he had done since he was taken ill. He had sent off his secretary to his army that morning, which was then on its march to Badajos, and intended to follow it, by easy journeys, next day himself.

I left him about eleven A.M. and at one was again called to him. An attack, similar to that of the 17th, had returned. He was supported in bed, and the perspiration was dropping from his forehead, from the violence of the pain. He referred it distinctly to one spot, a little to the right of the sternum. His breathing was not much affected. Though the pain was so violent, he said it was less intolerable than what he had experienced on the 17th. Fomentations were applied, and a small quantity of ether, with thirty drops of laudanum, given; but no good effects ensued. In about half an hour he became more disturbed, and suddenly fell back on his bed, and after three or four gasps expired.

The suddenness and mode of his death left no doubt that some disease existed about his heart or great vessels. With some difficulty I obtained permission to examine the body, which I did on the following day, in the presence of Mr. GUNNING, and of the Spanish physician who had resided in the family.

We found an aneurism of the aorta, about three inches beyond the semilunar valves, of the size of a pigeon's egg, filled with flakes of coagulable lymph. The artery had burst at the extremity of the sac into the pericardium; owing, apparently, to a sudden impetus of the blood, as no appearance of ulceration about the orifice could be perceived: it was merely a longitudinal fissure, one-fifth of an inch in extent, and the internal coat of the artery at its margin was smooth, and of a natural appearance. The coats of the vessel, where they formed the sac, were not a third of their usual thickness. Specks of ossification were met with in the artery, from its root to its arch. Through the orifice one pound and a half of blood had flowed into the pericardium, which was completely distended.

No other morbid appearances were discovered.

CASE II. *of Aneurism of the Aorta, terminating in Rupture into the Pericardium.* By Dr. HEWETT.

A. B—, a gentleman, apparently about forty years of age, had dined with some friends, and had left them in the evening, to return to his residence nof far from town. An alarm was given by a female that a gentleman was dying in the Park. He was conveyed to St. George's Hospital, but had expired before his arrival. It appeared that he had been taken suddenly ill, under circumstances of peculiar excitement.

*Sectio cadaveris*, October 29th, 1826.—The cavity of the pericardium was found distended with blood, which had escaped into it from an opening in a small aneurism, situated at the origin of the aorta, just within the portion of it covered by the pericardium. Another aneurism was found arising from the aorta, very near, but quite externally to the bag of the pericardium. The former aneurism scarcely equalled the size of an almond; the opening from the aorta into it was through a narrow irregular fissure in the inner membrane, of about a quarter of an inch in length, and co-extensive with the slightly elevated margin of a steatomatous deposition, under which there was a passage, in an oblique direction, into the little aneurismal pouch. The bursting of this aneurismal pouch had opened a communication into the cavity of the pericardium, and had caused instantaneous death. The second aneurism was larger and deeper; for it admitted the end of the thumb into the opening from the aorta, and protruded, forming a cul-de-sac of an inch and a half in depth, and of the size of a small walnut.

These aneurisms had obviously originated from some steatomatous depositions between the inner and fibrous coats of the aorta.

Though the neighbouring portion of the aorta was thickly studded with similar steatomatous formations, (which so frequently form the matrix for the deposition of particles of bony matter there,) still none of these had acquired the consistence of cartilage, much less the hardness of laminæ of bone. The inner membrane of the aorta, in the spaces between these yellow steatomatous depositions, was red, and highly vascular.

All the valves of the heart were in a perfectly natural condition. The brain also, and all the other organs, presented their natural appearances.

Case III. *Aneurism of the Aorta, which burst into the Œsophagus.*

Owen O'Brien, ætat. thirty-seven, a gardener, admitted at St. George's Hospital, May 2d, under the care of Dr. Young.

We are indebted to Dr. Hewett for the following information respecting this case also:

At the commencement of his illness, six weeks ago, he complained of pain in the left side of the head and general uneasiness, which he attributed to having caught cold. After a few days he had some difficulty of swallowing, and from the same date a slight cough. The difficulty of deglutition continued to increase for some time, till he was at last prevented from taking any solid food. No pulsation was at any time to be felt in the chest, though he several times complained of it. His pain was always referred to the right side of the sternum, and he was latterly unable to lie on his left side. Pulse about sixty, not intermitting. He died suddenly, being found dead in his bed by the nurse, on the morning of the 26th May, with blood issuing from his mouth.

On examining the body, an aneurism was found arising just below the point of departure of the left subclavian artery, and which had burst into the œsophagus. The stomach was distended with coagulated blood; and the air-tubes of the lungs also contained some, which appeared to have descended through the glottis.

---

HEMORRHAGE.

